# The Relationship Between Older Adults’ Attitude Towards AI and Likelihood of Using AI in Healthcare

**DOI:** 10.1093/geroni/igaf122.1470

**Published:** 2025-12-31

**Authors:** Amy Schuster, Gwendolyn Watson, Shubham Agrawal, Shelia Cotten

**Affiliations:** Clemson University, Clemson, South Carolina, United States; Auburn University, Auburn, Alabama, United States; Clemson University, Clemson, South Carolina, United States; Clemson University, Clemson, South Carolina, United States

## Abstract

Artificial Intelligence (AI) is changing the way many fields, including healthcare, operate. The adoption of AI depends on both technological innovation and users’ acceptance and use. Understanding factors that influence acceptance, like attitudes about AI, is critical for incorporating AI into healthcare. Older adults, compared to those younger, are more likely to have chronic medical conditions and use more healthcare services. We need to understand older adults’ attitudes about AI as they may be more likely to encounter AI used in healthcare. This study explored the relationship between older adults’ (60 years and older) attitudes towards AI (AI in general, human vs. AI interaction, and impact on well-being) and their likelihood of using AI in healthcare controlling for age, gender, race, level of education, relationship status, and self-reported health status. Data collected through an online survey (N = 405) were analyzed using multiple linear regression. Results indicate that older adults with more positive attitudes about AI in general and who were in more agreement that AI can have positive impacts on well-being were more likely to use AI in healthcare. Whereas older adults who had a higher preference of interacting with a human compared to an AI system were less likely to use AI in healthcare. Future research should explore factors contributing to older adults’ negative attitudes towards AI and design interventions to improve these attitudes and, thereby, increase their likelihood of using AI in healthcare.

